# Gene Therapy to the Retina and the Cochlea

**DOI:** 10.3389/fnins.2021.652215

**Published:** 2021-03-17

**Authors:** Ryan Crane, Shannon M. Conley, Muayyad R. Al-Ubaidi, Muna I. Naash

**Affiliations:** ^1^Department of Biomedical Engineering, University of Houston, Houston, TX, United States; ^2^Department of Cell Biology, University of Oklahoma Health Sciences Center, Oklahoma City, OK, United States; ^3^Oklahoma Center for Neurosciences, University of Oklahoma Health Sciences Center, Oklahoma City, OK, United States; ^4^College of Optometry, University of Houston, Houston, TX, United States; ^5^Depatment of Biology and Biochemistry, University of Houston, Houston, TX, United States

**Keywords:** retina, cochlea, gene therapy, nanoparticles, animal models

## Abstract

Vision and hearing disorders comprise the most common sensory disorders found in people. Many forms of vision and hearing loss are inherited and current treatments only provide patients with temporary or partial relief. As a result, developing genetic therapies for any of the several hundred known causative genes underlying inherited retinal and cochlear disorders has been of great interest. Recent exciting advances in gene therapy have shown promise for the clinical treatment of inherited retinal diseases, and while clinical gene therapies for cochlear disease are not yet available, research in the last several years has resulted in significant advancement in preclinical development for gene delivery to the cochlea. Furthermore, the development of somatic targeted genome editing using CRISPR/Cas9 has brought new possibilities for the treatment of dominant or gain-of-function disease. Here we discuss the current state of gene therapy for inherited diseases of the retina and cochlea with an eye toward areas that still need additional development.

## Introduction

Over 2.2 billion people worldwide experience vision impairment or irreversible vision loss^1^, and over 466 million people worldwide experience serious hearing loss^2^, making these the most prevalent sensory impairments. Many forms of vision and hearing loss are inherited and current treatments only provide patients with temporary or incomplete relief. Because many inherited sensory defects have identified disease genes, developing effective genetic therapies has been a primary research goal for several decades. Recent exciting advances in gene therapy have shown promise for the treatment of inherited retinal and cochlear diseases, both in the area of gene replacement and in the area of targeted genome editing (e.g., CRISPR/Cas9) to correct underlying genetic defects. However, there are still substantial limitations hampering widespread clinical use of genetic therapies in the retina and cochlea, including issues of distribution and uptake of the vectors by the target cells, a limited window of opportunity for treatment in degenerative disorders, attenuation of gene expression after a period of time, and efficiency/off-target concerns with novel genome editing strategies.

### Inherited Diseases of the Retina

**FIGURE 1:**
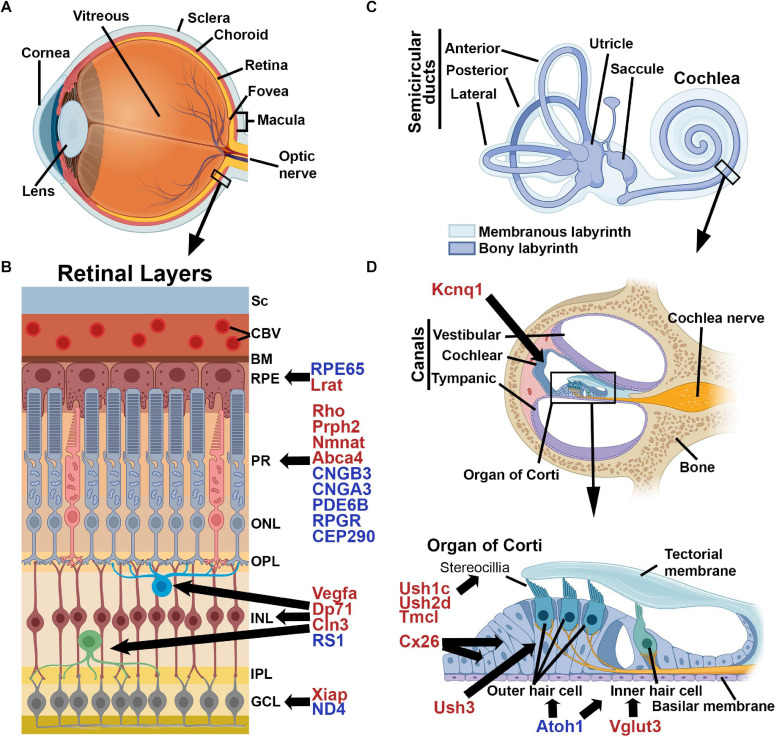
Schematic Illustrations of the Cochlea and Retina. **(A)** The structure of the eye, **(B)** layers of the retina (Sc, sclera; CBV, choroidal blood vessel; BM, Bruch’s membrane; RPE, retinal pigment epithelium; PR, photoreceptors; ONL, outer nuclear layer; OPL, outer plexiform layer; INL, inner nuclear layer; IPL, inner plexiform layer; GCL, ganglion cell layer) and **(C)** The structure of the inner ear, **(D)** cross-section of the cochlea and a close-up of the Organ of Corti. Shown are selected genes which are associated with disease in humans. Genes marked in red represent a selection of those that have been evaluated successfully in animal models while those in blue have been evaluated in human clinical trials. Images were created with BioRender.com.

The retina is the transparent, light-sensitive tissue at the back of the eye (Figure 1A) responsible for capturing light and converting it to an electrical signal to be delivered to the visual cortex in the brain where conception of vision occurs. The retina has evolved into a multi-cellular laminated structure, within which the different cells are organized and interconnected to maximize the efficiency and resolution of light perception (Figure 1B). The primary cells involved in the detection of light are the retinal rod and cone photoreceptors. Rods are necessary for low-light or “night” vision while cones perform bright-light color vision perception. The leading causes of irreversible vision loss are age-related eye diseases including age-related macular degeneration, diabetic retinopathy, glaucoma, and cataract^3^ (reviewed in Haddad et al., 2006; Simo-Servat et al., 2013; Singh and Tyagi, 2018). While these highly prevalent retinal diseases are not monogenic, they do often have a genetic component that contributes to disease in combination with environmental or other factors. In addition, inherited retinal dystrophies/diseases (IRDs) affect roughly 1 in 1380 individuals with an estimated 36% of the population as healthy carriers of one or more IRD causing mutations (Hanany et al., 2020). Combined, this underscores the need for the development of genetic therapies targeting the retina.

IRDs are a group of retinal dystrophies caused by mutations in over 271 different known genes (with many still unknown), that can target various cells within the retina. Virtually all inheritance patterns are represented in IRD genes including autosomal dominant, autosomal recessive, X-linked, digenic, polygenic, and even mitochondrial disorders. IRD genes expressed in photoreceptors can lead to various macular degenerations/dystrophies and retinitis pigmentosa (RP) and are involved in ocular development, phototransduction, cellular metabolism, and in the development and maintenance of photoreceptor structure. Mutations in retinal pigment epithelium (RPE) genes include those involved in retinoid recycling, cellular metabolism, and cellular homeostasis and can lead to macular dystrophies as well as Leber’s Congenital Amaurosis (LCA). Mutations in retinal ganglion cell IRD genes are largely associated with mitochondrial function, but also include regulators of intracellular transport and transcription, and can lead to optic atrophy. A summary of known IRD genes can be found at: https://sph.uth.edu/retnet/sum-dis.htm.

Depending on the gene, the onset of disease varies from birth to adulthood. Photoreceptor dystrophies are the most common within the retina and can be classified by the primary affected cell type and whether central or peripheral vision is lost first. RP and rod-cone dystrophies typically affect rods first, while macular dystrophies/degenerations and cone-rod dystrophies often affect cones first (Rattner et al., 1999). However, it is worth noting that in many cases of macular dystrophy, where macular cones are the first cells targeted, the causative gene is expressed in the adjacent RPE, with vision loss arising due to secondary cone damage. Patients with RP and rod-cone dystrophies often initially present with night-blindness which is followed by gradual peripheral vison loss (Ferrari et al., 2011). As the patients’ visual fields gradually shrink over time, irreversible vision loss eventually ensues (Ferrari et al., 2011). Individuals with cone-rod dystrophies have a rapid loss of central vision early in their life, which is followed by gradual peripheral vison loss (Morimura et al., 1998; Fukui et al., 2002; Klevering et al., 2004).

Current treatment options to correct the more mild symptoms of vision loss include corrective lenses or contacts and more permanent laser correction surgery. In more severe cases where progressive vision loss occurs due to retinal degeneration, a more invasive approach is necessary. These advanced therapeutic options are all still in development or in early clinical use and include gene therapy, gene editing, reprogramming, cell therapy, optogenetic therapies, and retinal prostheses. The time at which these therapies are useful varies greatly. For maximum efficacy, gene therapy needs to be delivered in the early stage of disease to prevent further degeneration. In contrast, other options have a wider therapeutic window, but have had less success thus far, both in development and in clinical use (Maeda et al., 2019; Wood et al., 2019).

### Inherited Diseases of the Cochlea

The inner ear is a small, fluid-filled compartment (Figure 1C) encased in the temporal bone and located at the base of the skull. The acoustic energy from sound is converted to an electrical impulse that the brain perceives as hearing through a multi-step process. Sound waves hit the tympanic membrane (eardrum) which vibrates the attached ossicular chain (three small ear bones). This vibration is transmitted to the fluid in the cochlea and basilar membrane (Figure 1D), and resulting fluid waves are sensed by the hair cells in the organ of Corti through mechanotransduction. The specialized sensory hair cells in the cochlea comprise a single layer of inner hair cells (IHCs) responsible for converting the mechanical signal into an electrochemical one, and three layers of outer hair cells (OHCs) involved in modulating and amplifying auditory signals. Mechanical deflection of stereocilia on the surface hair cells results in depolarization, and subsequent signal transmission to the auditory cortex (Rubel and Fritzsch, 2002). The differentiation of sound frequencies is facilitated by the length of the cochlea and the stiffness of the basilar membrane (Manoussaki et al., 2006).

Sensorineural hearing loss (SNHL), defined as the presence of a deafness-causing etiology in the cochlea or auditory nerve, accounts for more than 90% of hearing loss in adult patients. Approximately 50–60% of all cases of congenital deafness are directly linked to genetic factors, with higher numbers in developed countries (Marazita et al., 1993; Smith et al., 2005). Currently there are 49 identified genes known to be responsible for autosomal dominant non-syndromic hearing loss, 76 autosomal recessive genes, and 5 X-linked genes^4^ and (Duman and Tekin, 2012; Yuan et al., 2020) with many more remaining to be mapped. These genes play a role in a variety of processes including hair bundle development/function, hair cell adhesion, synaptic transmission, cochlear ion homeostasis, cellular homeostasis/energy generation, and regulation of the extracellular matrix (reviewed in Dror and Avraham, 2010; Stamatiou and Stankovic, 2013; Zhang W. J. et al., 2018). As in the retina, disease onset can vary from birth to late adolescence. The pattern and progression of symptoms in patients help identify what mutation(s) may be involved and determine whether interventions, genetic or otherwise, may be beneficial.

Current treatment options focus on amplifying sound through hearing aids or electrical stimulation of auditory neurons via cochlear implants for individuals with severe deafness. Cochlear implants are currently the most successful sensory prosthetics available, allowing deaf individuals to understand speech (Wilson and Dorman, 2008; Roche and Hansen, 2015). However, their effectiveness is limited to quiet spaces and suffers from poorly detailed sensory perception (Muller and Barr-Gillespie, 2015; Roche and Hansen, 2015; Weiss et al., 2016). In addition, while highly beneficial, cochlear implants merely treat the symptoms of hearing loss without addressing the underlying cause, so cochlear gene therapy has been an exciting therapeutic goal.

### Syndromic Sensory Inherited Diseases

**FIGURE 2:**
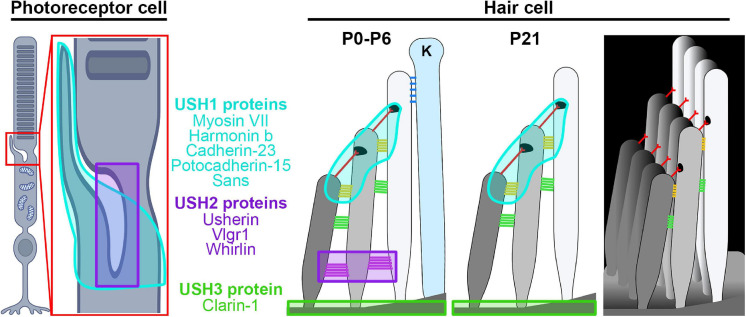
Site of Usher proteins in the retina and the cochlea. Shown are illustrations of the photoreceptor and hair cell highlighting the regions affected by different Usher syndrome genes. Ush1 genes/locations are shown in cyan, Ush2 genes/locations in purple, and Ush 3 genes in lime green. Hair cell legend structures: ankle links (purple), lateral links (green), contact region (gold), tip link (red), kinocilium links (blue), kinocilium (K). Images were created with BioRender.com.

In addition to inherited disease genes affecting a single tissue (e.g., the retina or the cochlea), there are a large variety of disease genes that are syndromic, affecting multiple tissues. Structural parallels in photoreceptors and cochlear hair cells mean that there are also a large number of disease genes that cause dual syndromic deafness and blindness. These genes often induce photoreceptor degeneration rather than optic atrophy and are frequently classed under the larger umbrella of ciliopathies. The most common form of dual deafness and irreversible vision loss involves the many genes associated with Usher syndrome (El-Amraoui and Petit, 2014; Mathur and Yang, 2015, 2019). Usher syndrome is clinically broken down into three types, Ush1, Ush2, and Ush3, due to the severity of hearing and vision loss, presence or absence of vestibular problems, and the age of onset for the symptoms (Millan et al., 2011). Patients with Ush1 are born with severe hearing loss, have abnormal vestibular systems, and progressive vision loss due to RP beginning during childhood. Individuals with Ush2 are born with mild to severe hearing loss and have progressive vision loss starting during adolescence. People with Ush3 develop hearing loss during adulthood and vision loss during adolescence and develop vestibular abnormalities. The types are further subdivided due to the underlying genetic cause. The proteins associated with each type are localized into similar regions in the retina and cochlea (Figure 2) and often associate or interact with one another, thus mutations in several genes can lead to similar patient phenotypes.

Other disease genes with multiple affected areas include many of the retinal disease genes that cause optic atrophy such as *MECR* and *ACO2* which are also associated with cerebellar defects and ataxias (Heimer et al., 1993; Sharkia et al., 2019). In other cases, these disorders also have a deafness component, for example in the case of *TIMM8A*, which is associated with deafness-dystonia-optic neuropathy syndrome (Tranebjaerg, 1993). However, these syndromic disorders are very complex, and in some cases, different mutations in the same gene lead to isolated optic atrophy or ataxias, such as the case of *AFG3L2* (Di Bella et al., 2010; Colavito et al., 2017). Some syndromic forms of vision loss such as Batten disease affect the brain and bipolar cells, the inner retinal neurons responsible for conveying signals from photoreceptors to ganglion cells (Kleine Holthaus et al., 2020). These various syndromic diseases highlight molecular similarities in various sensory disorders, and suggest that examining sensory disorders from a broader, non-organ-specific perspective may be beneficial.

### Commonalities Between the Retina and the Cochlea

#### Favorable Biological Characteristics

One problem with systemic delivery or delivery to systemic organs besides rapid clearance/excretion of the vector is potential activation of the immune system. In contrast, both the eye and the ear represent accessible local delivery sites that are fairly protected from the systemic immune system. This permits a prolonged half-life for delivered material and reduces the chance of a systemic immune response or off-target effects in other parts of the body.

The inside of the eye, including the retina, is isolated from the systemic circulation by the blood-retinal barrier and the blood-aqueous barrier, maintained by tight junctions between retinal pigment epithelial cells; between vascular endothelial cells in the ciliary processes, Schlemm’s canal inner wall endothelial cells and the iris vasculature; and between the cells in the non-pigmented ciliary epithelium (Campbell and Humphries, 2012; Coca-Prados, 2014). Thanks to these barriers, the eye has a limited immune system with only local immune and inflammatory responses present to maintain homeostasis, allowing the retina to be immune-privileged (Zhou and Caspi, 2010; Cunha-Vaz et al., 2011). Likewise, the cochlea is also isolated and surrounded by bone with even less access to the rest of the body. The endothelial cells lining the cochlear blood vessels form the blood-labyrinth barrier (BLB) or blood-cochlea barrier (BCB), which is comparable to the blood-brain barrier (BBB) in its ability to tightly regulate the exchange of macromolecules and ions between the blood system and the inner ear. The BLB maintains the homeostasis of the inner ear and isolates it from any pathogens found in the blood, allowing the inner ear to also maintain relative immune-privilege (Salt and Hirose, 2018).

Another benefit of genetic therapies for both the retina and cochlea is the need to deliver only small amounts of therapeutic agents, which is due to the ability to deposit material directly to or in very close proximity to the target cells, allowing for greater chance of uptake by the desired cells with limited loss due to systemic distribution.

In addition, the majority of the cells in both the retina and cochlea are post-mitotic and fully differentiated. This provides both opportunities and challenges for gene delivery. One substantial advantage is that the lack of cell division permits evaluation of changes in gene expression over time (for example due to vector loss or epigenetic changes) without concern about dilution of non-integrated vectors by cell division. Similarly, non-integrated vectors are not lost over time due to cell proliferation. In contrast, post-mitotic cells can be harder to transfect and target than dividing ones, making it more difficult to achieve cellular uptake of therapeutic vector. In addition, the non-proliferating nature of retinal and cochlear sensory cells means that in general once lost, cells are gone forever. Though research effort has been focused on combatting this by the use of stem cells or by regenerating cochlear hair cells, in general the degenerative nature of disease when post-mitotic cells are affected means the therapeutic window is narrow, as treatments delivered after too many cells have died will not be effective.

#### Testing and Development Benefits

From a therapeutic development standpoint, the bilateral nature of the retina and cochlea is also beneficial. Treating only one eye or cochlea allows the contralateral eye/cochlea to serve as an internal control for analysis, since the treatment rarely escapes the treated eye/cochlea. However, leakage from one ear to the other after scala tympani injections has been reported (Lalwani et al., 2002b), so restriction of therapeutic agents to a single eye or retina should be confirmed prior to interpreting resultant functional data.

Another benefit of developing and testing gene therapies in the retina and cochlea is that there are a variety of ways to perform non-invasive functional testing to assess therapeutic efficacy in both animal models and humans. The non-invasive nature of these tools also enables measurements to be performed longitudinally, essential for efficiently evaluating the duration of therapeutic effects. *In vivo* imaging in the retina includes spectral-domain optical coherence tomography (OCT) and fundus imaging/angiography to assess distribution of tagged genetic vectors and retinal structure. Non-invasive functional testing in the retina include electroretinography (ERG) to measure retinal neuronal responses, as well as optokinetic tracking (OKT) to measure visual acuity (Cai et al., 2010; Koirala et al., 2013). *In vivo* cochlear functional testing includes assessments of auditory brainstem responses (ABR) and distortion product otoacoustic emissions (DPOAE) to evaluate inner ear function. Imaging modalities can be used in parallel, including computed tomography (CT) and magnetic resonance imaging (MRI) to evaluate cochlear structure (Razek and Huang, 2012; Al-Rawy et al., 2017).

## The Use of Animal Models to Study Retinal and Cochlear Inherited Diseases

Preclinical gene therapy studies are an essential component of therapeutic development. They are used to assess safety and efficacy, but are also used to assess a variety of other parameters that can have a huge influence on outcomes. These can include (1) optimizing vector content, including promoter/enhance choice, gene sequence, inclusion of intronic and untranslated region (UTR) sequences, etc.; (2) evaluating methods to prevent gene silencing, such as elimination of CpG islands, the inclusion of insulators, etc.; (3) testing methods to promote or prevent genomic integration, such as the inclusion of transposases or S/MARs; (4) evaluating vector packaging, including the use or development of various viral compaction methods, engineering novel viral serotypes to enhance tissue tropism, engineering novel non-viral vectors; (5) assessing delivery methods; (6) assessing cellular uptake and targeting of vectors, including vector/particle optimizations to enhance cellular uptake; and (7) evaluating gene expression levels, knockdown efficiency, or duration of expression. The length of this list highlights how much development and testing often occurs before genetic therapies can even be evaluated for their ability to elicit functional improvement in the target disease.

Animal studies are necessary to fully understand the effects of gene therapy in a true organ microenvironment and to investigate the toxicities involved in long-term studies. The majority of preclinical retinal and cochlear studies have used rodents as preferred animal models. Mice have well characterized genomes, with genetic, biological, and behavioral characteristics similar to humans, and their genomes can be easily manipulated which is a boon for studies of inherited diseases. Guinea pigs have also been used for evaluation of auditory dysfunction as they are susceptible to noise induced hearing loss, making them a commonly used model for optimizing gene delivery approaches and to assess the efficacy of gene-based treatments not targeted to a specific disease gene (for example delivery of growth factors for general neuroprotection) (Chen et al., 2018; Pinyon et al., 2019; Lafond et al., 2020). Final preclinical studies for both safety and efficacy use larger animal models, including pigs, dogs, and non-human primates, as these animals may better mimic the anatomy or immune system of human structures or because they offer further evidence of efficacy, for example in the RPE65 dog model (Kelley et al., 2018; Maddalena et al., 2018; Ding et al., 2019; Gyorgy et al., 2019; Gardiner et al., 2020; Ivanchenko et al., 2020).

### Retinal Animal Models

There is an extensive body of literature using mouse models for retinal gene therapy (for example, there are over 1,700 PubMed hits for “mouse retinal gene therapy”) and for the study of IRD disease mechanisms (e.g., Sakamoto et al., 2009; Sakami et al., 2014; Chakraborty et al., 2020; Genc et al., 2020). However, there are some differences between the mouse and human eye that make evaluation in larger animals prudent. First, in contrast to the human retina which is developed at birth, the cells of the mouse retina are not fully produced prenatally. While the first wave of retinogenesis begins during embryonic stages (E11–E18), including horizontal cells, cone photoreceptors, some amacrine cells, and retinal ganglion cells, the second wave of retinogenesis takes place postnatally (P0–P7) with the rod photoreceptors, bipolar interneurons, and Müller glial cells (Cepko et al., 1996; Heavner and Pevny, 2012). Thus studies showing effective gene therapy after early postnatal treatment in mice may not translate well to humans. There are also structural differences in addition to the obvious size difference, most notably the larger relative size of the lens and the lack of a macula in mice. The flatter lens in human eyes (compared to mice) means that the relative intravitreal volume is quite different, critical for designing and evaluating dosing strategies. Also important is the lack of a macula in the mouse eye. The macula is both very fragile and the primary site of pathology for many IRDs, so the lack of a macula in the mouse can make it challenging to test therapies that need to target the macula or adjacent areas. In addition to lacking a macula, the rod:cone ratio in mice is much higher than in humans, making studies on cone-specific macular dystrophies more challenging (Volland et al., 2015). In spite of this, many successful preclinical studies (further discussed below) have used mice to assess gene therapies in models of cone or macular dystrophies, however, modeling the physical constraints of the macula is challenging in rodents. Testing gene therapies in non-human primates allows for a better approximation of how results will translate clinically, especially with regards to vector delivery, uptake, and gene expression (Yiu et al., 2020). Non-human primates also have a macula, which is advantageous for developing treatments for both IRDs and other macula-targeted disease such as age-related macular degeneration (Picaud et al., 2019). However, there are several limitations to using non-human primate models, including cost, ethical concerns, and perhaps most importantly, the lack of models for IRDs. One rare early exception to the lack of non-human primate genetic models was a squirrel monkey model in which trichromatic vision was restored to animals lacking the L-opsin gene by the delivery of an adeno associated virus (AAV) 2/5 vector (Mancuso et al., 2009). However, in general, functional efficacy of IRD treatments has not been evaluated in non-human primates due to the historical lack of genetic flexibility.

Slowly however, there are signs that this lack of genetic primate models may be changing due to the genome-editing capabilities of CRISPR-based genome editing strategies. Germline knockout models in non-human primates have been established (Niu et al., 2014), but the long life-span and singleton birth pattern make germline models in non-human primates practically prohibitive. More recently however, a somatic, macular knockout model of *CNGB3*-associated achromatopsia has been generated in monkeys using AAV9-mediated delivery of a CRISPR/Cas9-based construct (Lin et al., 2020). Similarly, somatic, AAV5-mediated delivery of a CRISPR/Cas9 *GUCY2D* knockdown construct was used to generate a macaque model of cone-rod dystrophy (McCullough et al., 2019). While still in the early stages, these innovative genome editing tools may pave the way for expanded use of large animal models.

### Cochlear Animal Models

Mice are also widely used as genetic models for hearing loss. Mouse models of hearing loss have been created using a variety of genetic approaches, ranging from traditional knockouts to modern gene-edited germline models (see for example Charizopoulou et al., 2011; Michel et al., 2017; Schrauwen et al., 2018; Zhang H. et al., 2018). The major difference between the mouse and human auditory system is the timeline of development. Mice are naturally born deaf and don’t begin to hear until ∼P14, as the final development of the organ of Corti occurs early postnatally. This is a practical disadvantage for gene therapy for congenital deafness, firstly because delivery in rodent models during the early postnatal period—while likely to be successful—would be difficult to mimic clinically since the corresponding human developmental stage occurs *in utero*. Secondly, it has been proposed that in many cases of congenital deafness the cellular and molecular disease process has already started *in utero*, making effective postnatal interventions all the more difficult to achieve. While mice are widely used in genetic studies, guinea pigs are more notably used in cochlear studies involving cochlear implants due to easier and more reproducible surgical access to the inner ear compared to other rodent models (Wysocki, 2005; DeMason et al., 2012; Mistry et al., 2014) and their susceptibility to noise-induced hearing loss. The guinea pig also has a hearing range which is more conducive to human hearing comparisons, compared to other small rodent species (Heffner and Heffner, 2007).

In addition to developmental differences, there are also anatomical differences between the mouse and human ear that are relevant to the evaluation of gene therapies. The human cochlea and labyrinth are approximately 20-fold larger than rodents and 3 to 4-fold larger than monkeys (Ren et al., 2019). Studies have found that inner ear volumes correlated with animal body mass across different species while looking at mice, rhesus monkeys, and humans (Ekdale, 2013; Dai et al., 2017) suggesting that the larger size of primates might enable them to serve as intermediate models for translation of rodent findings into humans. In addition to inner ear volume differences, there are also anatomical differences between the human and mouse ear that have implications for gene/drug delivery. Intratympanic delivery is a common way to deliver drugs destined for the inner ear in patients and rodent models, and it is thought that drugs have some permeability through the otic capsule (the bony structure surrounding the cochlea) in addition to through the round window membrane. However, the human otic capsule is significantly thicker than that in mouse and guinea pig, so human drug/therapeutic delivery throughout the length of the cochlea may not be well-modeled by rodent studies (Mikulec et al., 2009).

Assessments of audiovestibular function after saline injection into the oval or round window in non-human primates showed no evidence of toxicity as assessed by auditory function, histology, or behavior suggesting that drugs and therapies can be delivered to the primate ear (Dai et al., 2017). Consistent with this observation, primate models have been used to evaluate genetic therapies; for example, transmastoid injection in the round window of AAV9 carrying a GFP reporter achieved >90% transduction of both inner and outer hair cells in one of their test animals but no clear transduction of hair cells in the other (Gyorgy et al., 2019). In addition, as with the retina, the lack of genetic models for hearing loss in primates has also prevented their widespread use for testing of therapeutic efficacy, and as yet, no CRISPR/Cas9 non-human primate models of SNHL have been reported.

## Delivery Methods for Gene Therapy

### Delivery of Genes to the Retina

**FIGURE 3:**
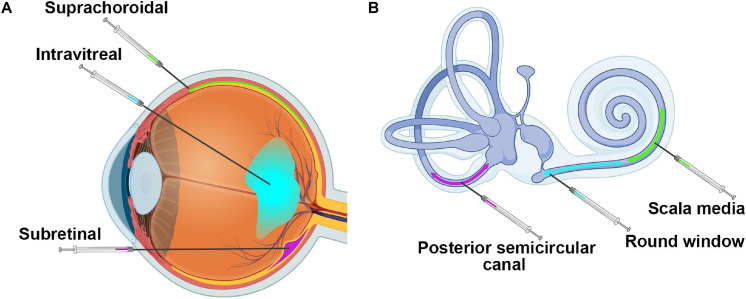
Injection sites into the retina and the cochlea. Shown are **(A)** the three major injection sites for delivery to the retina and **(B)** the three main injection locations for the cochlea. Images were created with BioRender.com.

Delivery of genetic therapies to the retina typically involves subretinal, intravitreal, or suprachoroidal injection of material, with variations on the site of entry (Figure 3A). Subretinal injections deliver material between the photoreceptors and the RPE. This is the most widely used method for therapies targeting photoreceptors or RPE since material is in close proximity to most target cells. However, the method is invasive, causing retinal detachment at the site of injection, and has greater potential for complications during the procedure. Intravitreal injections avoid these issues by delivering into the vitreous cavity of the eye. This procedure is in routine clinical practice for the delivery of anti-angiogenesis agents to treat neovascularization associated with diseases such as age-related macular degeneration (Todorich et al., 2014). However, material delivered intravitreally typically has poor penetration into the outer retina (Farjo et al., 2006; Dias et al., 2019), due both to the content of the vitreous itself as well as to the inner limiting membrane which forms a physical barrier between the retina and the vitreous. Identifying ways to improve delivery of genetic material to the outer retina after intravitreal delivery has been a critical research goal in the past several years. Many methods have been tried, including making genetic modifications to AAV capsids (Petrs-Silva et al., 2009; Dalkara et al., 2013), including agents to digest or disrupt the inner limiting membrane (Dalkara et al., 2009; Cehajic-Kapetanovic et al., 2011), application of electrical currents (Song et al., 2019, Song C. et al., 2020), and the inclusion of adjuvants such as tyrosine kinase inhibitors or proteasome inhibitors (Dias et al., 2019). Modification of viral capsids led to almost complete transduction of the outer retina after intravitreal injection in the mouse retina, but did not achieve this milestone in larger animal models such as dog and non-human primate (Mowat et al., 2014; Boyd et al., 2016; Ramachandran et al., 2017). This highlights an area where testing in larger animals is essential due to the relative (and absolute) difference in vitreous volume and inner limiting membrane thickness between mice and other larger animals (Matsumoto et al., 1984; Puk et al., 2006; Dalkara et al., 2009). Consistent with this, many intravitreal optimization studies are now being done in large animals, including sheep and non-human primates (Ross et al., 2020; Song H. et al., 2020), and overall this field has made significant advancements. While all of the above-mentioned studies were designed to optimize delivery of AAV-based gene therapies to the outer retina, researchers are also experimenting with ways to enhance non-viral gene delivery to the outer retina after intravitreal delivery. For example, various agents, such as chloroquine and hyaluronic acid, have been incorporated into non-viral delivery complexes in order to enhance uptake, but thus far they have met with limited success (Devoldere et al., 2019; Mashal et al., 2019).

More recently, suprachoroidal injection has gained prominence for gene delivery to the outer retina. In this method, material is delivered between the choroid and the sclera (Figure 3A), a region largely accessible only in larger animals. It is further complicated by the need for advanced imaging techniques, such as OCT, to visualize and inject through the use of novel microneedles (Patel et al., 2011; Kim et al., 2014; Yiu et al., 2014; Willoughby et al., 2018). The most notable advantage of this approach is the potential ability to reach the outer retina without performing a disruptive subretinal injection, and many groups are exploring this new avenue. Recently, a phase 3 clinical trial using suprachoroidal injection to deliver triamcinolone for the treatment of uveitis with macular edema reported no serious adverse events related to the treatment and positive efficacy outcomes, paving the way for future clinical use of this delivery method (Yeh et al., 2020). Various groups are testing the use of this method for retinal gene therapy with AAVs in a variety of animal models including rats, pigs, and non-human primates with generally promising results (Touchard et al., 2012; Ding et al., 2019; Yiu et al., 2020). Some groups have also been testing suprachoroidal delivery with non-viral methods, but few full published results are available yet (Kansara et al., 2020). The suprachoroidal approach has great promise for less invasive delivery to the retina, but there are concerns about penetrating through multiple layers of tissue (from the choroid through the RPE into the retina) and about initiating immune responses. One alternative approach that has been shown to induce better retinal transduction with attenuated immune responses in non-human primates is transscleral microneedle-based subretinal delivery (Yiu et al., 2020), which may be another delivery method for further exploration.

### Delivery of Genes to the Cochlea

As the cochlea is encased in bone (Figure 1C), the delivery of material to the right location without creating significant damage that may lead to further hearing loss is a major challenge. Largely because of this delivery challenge, the field of cochlear gene therapy has been slower to develop than retinal gene therapy. Yet in the last few years, preclinical studies targeting the cochlea have rapidly increased in number, as tools and methodologies have improved. Modes of delivery for the cochlea involve cochleostomy in the lateral wall of the cochlear basal turn, canalostomy in the semicircular canal or injections through the round window, scala media, or posterior semicircular canal (Figure 3B). Cochleostomy, in which a hole is drilled in the basal cochlea into the endolymphatic space and material is thereby injected directly into the scala media, has been shown to be reasonably efficient in generating AAV-mediated gene expression in the inner ear (Shibata et al., 2009; Yoshimura et al., 2018). However, the procedure has been shown to have higher potential to induce hearing loss than round window membrane injection (Chien et al., 2015; Delmaghani and El-Amraoui, 2020). Round window membrane injections have been successfully used in early postnatal mice (Akil et al., 2012; Askew et al., 2015; Landegger et al., 2017; Pan et al., 2017), and very recent studies have shown this method to be effective in adult non-human primates (Gyorgy et al., 2019; Ivanchenko et al., 2020). Others have shown reporter gene or therapeutic gene expression in the murine inner ear after *in utero* injection of AAV2 into the otocyst (Miwa et al., 2013; Hu C. J. et al., 2020), potentially important given the very early onset of many forms of congenital deafness. Canalostomy involves injection in the posterior semicircular canal, and has been shown to efficiently transduce inner and outer hair cells after treatment of adult mice and is reported to have less chance of leading to damage (Suzuki et al., 2017). Canalostomy can also be combined with round window membrane injection to further improve inner ear transduction in adult mice (Yoshimura et al., 2018). While much remains to be refined in the area of cochlear gene delivery, recent successes using non-human primate models support an optimistic view for the future of human cochlear gene delivery.

## Gene Therapy in the Retina and Cochlea

### Preclinical Gene Therapy in the Retina

The earliest proof-of-principle studies for retinal gene therapy took place in the early 1990s and involved gene supplementation via transgenesis in spontaneously occurring IRD models (Lem et al., 1992; Travis et al., 1992). While paving the way for several decades of further development, even these early studies highlighted one of the most challenging, and as yet incompletely resolved, issues in retinal (and cochlear) gene therapy: levels of expression. For both recessive IRD genes as well as autosomal dominant genes associated with haploinsufficiency, generating enough expression to mediate full rescue remains problematic (Ali et al., 2000; Cai et al., 2009). Shortly after these initial transgenesis studies, efforts were made to use adenovirus to transduce the retina (Bennett et al., 1994; Li et al., 1994), but safety concerns and lack of efficiency led to a shift to the use of AAV (Ali et al., 1996). More recently, lentivirus has been used for retinal gene therapy, especially for targeting neovascularization, but lentiviruses have thus far been fairly ineffective at targeting photoreceptors (Kalesnykas et al., 2017; Becker et al., 2018; Holmgaard et al., 2019). In the intervening 25 years, AAV-mediated retinal gene therapy had been used in a vast number of different preclinical IRD models, including those targeting photoreceptors (e.g., *Rho*, *Prph2*, *Nmnat*, *Abca4*, *Cngb3, Rpgr)*, retinal ganglion cells (e.g., *Xiap)*, Müller cells (e.g., *Vegfa, Dp71)*, bipolar cells, (*Cln3)*, and the RPE, (e.g., *Rpe65, Lrat*) (Ali et al., 2000; Batten et al., 2005; Mookherjee et al., 2015; Georgiadis et al., 2016; Becker et al., 2018; Zhang et al., 2019; Barboni et al., 2020; Greenwald et al., 2020; Kleine Holthaus et al., 2020; McClements et al., 2020; Wassmer et al., 2020; Figure 1B). Notable successes with these models led to testing in larger animal models, in particularly RPE65 and RPGR dog studies providing further support for the efficacy of AAVs (Bainbridge et al., 2015; Beltran et al., 2015; Gardiner et al., 2020; Song C. et al., 2020).

Much recent work in the field of retinal gene therapy with AAVs has focused on evolving viral capsids to improve transduction efficiency and refine the type of cells that can be targeted. This has been successful in the mouse, where novel engineered viral capsids have been shown to be much more efficient that parent viruses (Dalkara et al., 2013; Khabou et al., 2016; Frederick et al., 2020). Because viral tropsims can also be species specific, recent work on directed evolution of AAV capsids has used the non-human primate model (Byrne et al., 2020). This approach involves injection with libraries generated with *cap* gene variants, harvesting the retina, performing PCR to enrich *cap* gene variants present (i.e., that transduced cells) and reformulating virus. This selection process is repeated several times with additional steps included in subsequent rounds to increase diversity in *cap* genes. The exciting end result was the identification of novel AAV variants capable of efficiently transducing non-human primate cones after intravitreal injection, a key therapeutic goal that had not been achieved previously (Byrne et al., 2020).

While AAV-mediated therapies have been remarkably successful, there have been some concerns. Though largely considered safe, some studies have shown that the AAV vector is able to move from the eye to other areas of the body, from both intravitreal (Dudus et al., 1999; Han et al., 2012) and subretinal injection (Weber et al., 2003). Along with potential systemic distribution and immunogenicity, another challenge with AAV is its loading capacity. Standard AAVs can only accommodate genes which are <5 kbp (Wu et al., 2010), smaller than many IRD genes such as *USH2A* and *ABCA4*. There have been advancements in AAVs which increase capacity to 10 kbp, through dual AAV vectors. Dual AAV vectors split the load into two fragments which are then co-delivered and recombine in the target cell though either homologous recombination or trans-splicing strategies (Xu et al., 2004; Ghosh et al., 2011; Trapani et al., 2014). Though initially the efficiency of this approach was limited, additional novel strategies to overcome size limitations are being studied including the use of oversized AAV, trans-splicing dual AAV, overlapping dual AAV, and hybrid dual AAV (reviewed in Trapani, 2019). However, as a result of these potential limitations, for many years there has been interest in the development of non-viral gene delivery methods.

Though many different non-viral formulations have been successfully used to transfect cells, fewer have been successful *in vivo.* One of the most successful has been polylysine DNA nanoparticles complexed with polyethylene glycol. These nanoparticles are non-immunogenic, and have the capacity to compact up to 20 kbp of DNA (Fink et al., 2006). Importantly, they have demonstrated robust transfection, sustained for up to 15 months, in both photoreceptors and the RPE in the mouse retina (Farjo et al., 2006; Han et al., 2012; Koirala et al., 2013). This translated into improved functional outcomes in *Prph2*, *Rho, Abca4*, and *Rpe65*-associated IRD models (Cai et al., 2009, 2010; Koirala et al., 2013; Han et al., 2014, 2015). Other non-viral retinal gene therapy formulations have also recently been shown to promote improvement in murine IRD models. For example, solid lipid nanoparticles carrying the human *RS1* gene have been shown to mediate structural improvement in the mouse *Rs1h* knockout model of X-linked juvenile retinoschisis (Apaolaza et al., 2015, 2016). Another lipid-based system has also recently shown promising results. The ECO-lipid ((1-aminoethyl) iminobis[N-(oleicylcysteinyl-1-amino-ethyl) propionamide]) based system incorporates a pH sensitive endosomal escape mechanism to help promote release and preservation of DNA after uptake by cells. This system is multifunctional and can be conjugated to other compounds to facilitate targeted cell uptake. It has recently been used to deliver plasmid DNA and mediate improvements in the *Rpe65* and *Abca4* IRD models (Sun et al., 2017, 2020).

The vast majority of IRD model recue studies have involved gene supplementation to treat loss-of-function or haploinsufficiency-associated mutations. However, many IRD gene mutations are gain-of-function or dominant-negative alleles and for many years researchers have recognized the need for more complex therapies capable of knocking down mutant genes while also supplementing with the correct allele. This approach has been tested in a dog model of adRP; a mutation-independent shRNA targeting rhodopsin was paired with an RNAi-resistant human *RHO* cDNA (in AAV), and shown to promote long-term protection of retinal structure and function (Cideciyan et al., 2018). Proof-of-principle studies using RNAi-based approaches to knock down mutant transcripts have also been evaluated in mice for the treatment IRD associated with *Prph2*, *Impdh1* (RP10), *Guca1a* (cone-rod dystrophy), and *Pde6b* (RP) with varying levels of success (Tam et al., 2008; Tosi et al., 2011; Petrs-Silva et al., 2012; Jiang et al., 2013).

More recently, CRISPR/Cas9 based genome-editing approaches have gained popularity in preclinical studies to correct dominant IRD mutations. These approaches hold great promise for somatic gene editing, and various approaches have been tried. Perhaps the most obvious strategy is correcting IRD mutations using homology directed or micro-homology repair-based approaches. This has been tried in an RPGR model carrying a Cas9 transgene. AAV2/8 was used to deliver an RPGR-targeted gRNA and a region of homologous DNA carrying the corrected sequence (Hu S. et al., 2020). This study reported sequence correction in a large percentage of cells but the benefits were only apparent after ∼6 months, likely due to the overall inefficiency of homology directed repair. In addition, a challenge with homology-based repair approaches is the small carrying capacity of AAV. Designing a construct for delivery of Cas9, a homologous DNA segment for repair, and the gRNA can be challenging. To overcome this challenge, other groups have used two separate AAVs, one carrying the Cas9 and one carrying the gRNA and homologous region. This approach was used to target *Rpe65*, and reported homology-directed repair efficiencies in excess of 1%. Though this seems low, it was sufficient to mediate functional improvement. Other groups have taken advantage of an alternative repair pathway called micro-homology-mediated end joining, where a much shorter (∼20 bp) homologous region is supplied, and the whole expression cassette (Cas9, gRNAs, and homology region) fit into a single AAV. A study using this approach to target a *Gnat1* model reported improvement in light-sensitivity and visual acuity (Nishiguchi et al., 2020). An alternative approach for gain-of-function mutations is to target the mutant allele for knockout rather than correcting the mutation. While genes associated with haploinsufficiency may require concurrent gene supplementation, this approach has the benefit of relying on more efficient non-homologous end joining mechanisms rather than on the less efficient homology-based repair mechanisms. It has been tried with success in both rat and mouse models of rhodopsin-associated IRD (Bakondi et al., 2016; Giannelli et al., 2018; Patrizi et al., 2021). In addition to using CRISPR/Cas9 for gene editing, there are several alternative approaches that utilize a dead (d)Cas9/gRNA to target a transcriptional regulator to the appropriate place in the genome. A recent exciting study used this approach to target the VPR transcriptional activator (dCas9-VPR) to the M-opsin gene (*Opn1mw*) with an *Opn1mw*-specific gRNA where it activates gene transcription (Bohm et al., 2020). When dCas9-VPR/*Opn1mw*-gRNA under the control of the rhodopsin promoter was delivered to the *Rho*^±^ retina, researchers observed expression of M-opsin in rods, and importantly, improvements in scotopic vision and retinal structure. Genome editing in the mouse retina has also been attempted for *Pde6b*-associated RP (using electroporation), *Cep290*-associated LCA (further discussed below), and *Gucy2d*-associated cone-rod dystrophy (Ruan et al., 2017; McCullough et al., 2019; Vagni et al., 2019). Somatic genome-editing, and non-genome editing CRISPR-based therapeutic strategies are likely to become increasingly prevalent, particularly as tools to block chronic Cas9 activity, suppress immune responses, and increase the efficiency of homology directed repair are developed (reviewed in Nakamura et al., 2021).

The earliest clinical retinal gene therapy trials used AAV2 based vectors to deliver *RPE65* for the treatment of LCA (Bainbridge et al., 2008; Cideciyan et al., 2008; Maguire et al., 2008), with groups reporting varying degrees of improvement and persistence of effect. In initial phase I/II clinical trial using the AAV2/2 vector carrying *RPE65* cDNA with an RPE65 promotor, investigators observed improved visual function for 3 years (Bainbridge et al., 2008, 2015). Another group using rAAV2 carrying *h*RPE65 driven by the ubiquitous, chicken β-actin (CBA), promotor found no significant side-effects after 12 months but reported no significant improvement in vision (Hauswirth et al., 2008; Cideciyan et al., 2009). A follow-up done 6 years later saw progressive vision loss, reduced retinal sensitivity, and thinning of the photoreceptor outer nuclear layer (Jacobson et al., 2015). The third initial trial was conducted by Spark Therapeutics Inc. (Philadelphia, PA) using AAV2-hRPE65v2 with a CBA promotor (at three different doses) and reported sustained improvement after 2 years regardless of the dosage, with the greatest improvement in the younger children (Maguire et al., 2008, 2009). A few years later Spark Therapeutics Inc. released its results from a phase 3 clinical trial data using Luxturna^TM^ (voretigene neparvovec, AAV2-hRPE65v2) in patients with vision loss associated with RPE65. Bilateral subretinal injections of the highest dose tested in the prior trial found improved vision with no adverse effects or immune response after 1 year (Russell et al., 2017). This is the first phase 3 clinical trial to report significant gene therapy efficacy for IRD, and after submission to the FDA, Luxturna was approved for use and is now in clinical practice.

Since then, several other clinical trials for retinal gene therapy have been undertaken (Figure 1B). Recently promising results from a PhaseI/II trial for *CNGA3*-associated achromatopsia were published showing that the AAV vector was well-tolerated and initial measures of efficacy were promising, including improvement in visual acuity, contrast sensitivity, and cone function [NCT02610582 (Fischer et al., 2020)]. Many other retinal gene therapy clinical trials are currently ongoing or recruiting, targeting *RPGR* and *PDE6B*-associated RP, *CNGA3* and *CNGB3*-associated achromatopsia, *RPE65*-associated LCA, *RS1*-associated x-linked retinoschisis, and *ND4*-associated optic neuropathy^5^. All of these studies utilize an AAV-based delivery method, and thus far non-viral retinal gene therapy has not yet progressed to clinical testing. Excitingly, the first retinal gene therapy trial to use CRISPR/Cas9-based genome editing has recently started recruiting (NCT03872479). The trial, which targets *CEP290*-associated LCA is not only a milestone in retinal gene therapy, but also represents the first time CRISPR-based treatments will be delivered directly into the body in any trial, and results are eagerly anticipated. This study uses an AAV5 vector to deliver Cas9 and *CEP290* specific gRNAs and was effective in both a humanized *CEP290* mouse model and in a non-human primate model (Fischer et al., 2020).

### Cochlear Gene Therapy

As in the retina, the earliest cochlear gene therapy took the form of proof-of-principle studies utilizing transgenic overexpression to rescue hearing in mutant models with hearing loss (Fujiyoshi et al., 1994; Ahmad et al., 2007). By the late 1990s, researchers were exploring ways to deliver AAVs and adenoviruses to the middle and inner ear often using the guinea pig model described above (Lalwani et al., 1996, 1998; Mondain et al., 1998). Subsequently, an extensive number of functional studies were undertaken to genetically deliver antioxidants or neurotrophic factors to the ear for the treatment or prevention of noise- and pharmaceutical-induced auditory toxicity, rather than for inherited cochlear diseases (e.g., Bowers et al., 2002; Lalwani et al., 2002a; Kawamoto et al., 2003b, 2004; Liu et al., 2008). However, neither of these approaches targets or corrects the underlying genetic defect in the case of inherited forms of hearing loss. Excitingly, in 2012 a model of congenital deafness was rescued by delivery of a gene therapy vector. This landmark study used an AAV1 vector delivered by either round window membrane injection or cochleostomy to generate Vglut3 expression in a *Vglut3* knockout mouse model of hereditary deafness. This study demonstrated almost complete rescue of ABR recordings for over one year (69 weeks was the longest time evaluated) after delivery at P10-12 (Akil et al., 2012). Subsequently, antisense oligonucleotides were used to correct splicing in an Usher syndrome mouse model (*Ush1c*) leading to functional improvements in low-frequency hearing (Lentz et al., 2013; Ponnath et al., 2018; Wang et al., 2020). Since then, some AAV-mediated functional and/or structural rescue has been reported in multiple inherited models of deafness, including those associated with mutations in the gap junction gene connexin 26, the potassium channel subunit *Kcnq1*, the stereocilia gene whirlin, the antioxidant gene *Msr3b*, the usher 1C gene harmonin, the usher 3 gene clarin, the gene *Slc26a4*, and the ion channel *Tmc1*, among others (Yu et al., 2014; Chang et al., 2015; Iizuka et al., 2015; Chien et al., 2016; Kim et al., 2016; Pan et al., 2017; Dulon et al., 2018; Nist-Lund et al., 2019; Lan et al., 2020; Figure 1D).

Delivery of therapeutics to neonatal mice allows for therapeutic intervention before the organ of Corti develops fully, thus allowing for administration before early postnatal degeneration has begun, and this approach has been taken by several groups (Landegger et al., 2017; Pan et al., 2017). For example, AAV-mediated expression of *Ush1g*, a submembrane scaffold protein necessary for morphogenesis of the sensory stereocilia in cochlear hair cells, was delivered into *Sans* null mutant (*Ush1g^–/–^*) mice at P2.5 and found to preserve hair cell function near wild-type levels (Emptoz et al., 2017). Critically, studies such as this one demonstrate that cochlear AAV-mediated gene delivery can generate protein that folds, transports, and functions properly, and that early delivery prior to full development can prevent hair cell degeneration.

Delivering AAV to older/adult mice (compared to neonates) results in a drop in efficiency and duration of expression. For example, AAV-mediated delivery of *Vglut3* led to better and longer lasting recovery after neonatal delivery (P1-3) than later delivery (P10) (Akil et al., 2012), although good results were reported for P10-12 delivery as well. A more recent investigation using a modified AAV2 vector to deliver harmonin at either P0-1 or P10-12 found improvement in the function and structure of the P0-1 treated mice with no treatment effects in those treated at P10-12 (Pan et al., 2017). However, other recent studies have achieved good gene expression in hair cells without damaging the organ of Corti after delivery in adult animals, with delivery at either 6 or 10 weeks of age (Suzuki et al., 2017; Tao et al., 2018; Yoshimura et al., 2018). Although gene expression was lower in most cases than with neonatal delivery, it is encouraging that post-development delivery for cochlear therapy is becoming more effective.

As in the retina, there has been great interest in designing optimized AAVs for cochlear delivery. This includes several new synthetic AAVs such as Anc80L65, which has shown very high efficiency in transducing both inner and outer hair cells after injections at 7 weeks of age through the posterior semicircular canal (Suzuki et al., 2017; Hu C. J. et al., 2020). As in the retina, the limited capacity of AAV (<5 kbp) is a significant limitation. This is particularly problematic since many cochlear disease genes, such as those associated with Usher syndrome, are too large to be delivered by AAV. As a result, the dual-AAV strategy, in which one AAV carried the 5′ end of the gene (in this case the congenital deafness gene otoferlin), while the second AAV carried the 3′ end has also been evaluated in the cochlea. Although this requires recombination of the two cDNAs after delivery, researchers reported stable otoferlin gene expression and improvement in hearing after dual-AAV delivery, suggesting it may be a useful therapeutic approach (Akil et al., 2019).

A number of non-viral gene therapy strategies have also been tested in the cochlea. One of the earliest was a study in which cationic liposomes were microinjected into the guinea pig cochlea, and short-term reporter gene expression was observed in the organ of Corti (Wareing et al., 1999). Lipid core nanocapsules have been evaluated for their ability to penetrate through the round window membrane for subsequent material delivery (Zou et al., 2008). Polyethyleneimine (PEI)/DNA complexes and quaternized chitosan coupled Na-carboxymethyl-beta-cyclodextrin complexes have also been tested for their ability to generate reporter gene expression, but overall transfection efficiency still has room for improvement (Tan et al., 2008; Ren et al., 2010).

CRISPR/Cas9 and somatic genome editing strategies have also begun to be applied to the cochlea. One major potential benefit of this approach is that if the endogenous gene error can be corrected in enough cells, the challenge of generating sufficient levels of expression from an exogenous transgene is circumvented. While some groups are working on optimizing individual steps in the path to effective *in vivo* cochlear genome editing (Tang et al., 2016; Kang et al., 2020; Zhao et al., 2020), other groups have directly applied genome editing technology to models of inherited deafness. Gao et al. used cochleostomy to deliver a cationic liposome formulation containing a Cas9-gRNA ribonucleoprotein (RNP) complex targeting a mutant *Tmc1* allele to the scala media at P0-2. They reported improved preservation of hair cells at 8 weeks post-injection and improved ABR thresholds at 4 weeks post-injection compared to uninjected controls (Gao et al., 2018). These exciting preliminary studies demonstrate the potential for future application to somatic genome editing for cochlear diseases.

In spite of rapid advancements in preclinical testing of cochlear gene therapies since 2012, this strategy has not made the jump to widespread clinical testing. Only one gene therapy Phase I/II safety and tolerability clinical trial for severe hearing loss has been completed (NCT02132130). The study involved intralabyrinthine delivery of recombinant adenovirus carrying the Hath1/Atoh1 transcription factor (Figure 1D). This transcription factor has been widely evaluated in preclinical studies to promote hair cell regeneration, however few data are available about the trial and its findings have not yet been reported, although as of September 2020, the study is listed as “completed” and results are eagerly awaited (Crowson et al., 2017).

## Role of Time of Delivery and Duration of Effect on the Outcomes

While progress is being made for both retinal and cochlear gene therapy, it is necessary to remember one of the key limiting factors of gene therapy for inherited dystrophies: time. Due to the nature of degenerative hereditary diseases, unless intervention is applied early, the optimal therapeutic window will be missed. In many cases patients may not even know they have a genetic disorder until symptoms are severe enough to see their physician, at which point, the progression of the disease is often far enough along that affected cells are dead or dying. This is particularly challenging in the case of degenerations that affect neuronal cells, since there is, as yet, no effective way to regenerate or replace lost neurons (although this is a goal of the stem cell transplantation field). To a certain degree, this is an area where cochlear gene therapy is ahead of retinal gene therapy. There have been extensive preclinical studies focused on regeneration of hair cells in deafened mice (largely in non-genetic models) by delivery of the transcription factor Math1 (also known as Hath1/Atoh1) (Kawamoto et al., 2003a; Shou et al., 2003; Baker et al., 2009; Kraft et al., 2013), and several of these studies have produced promising results. However, while cochlear regeneration of hair cells may be more within reach than photoreceptor regeneration, the earlier onset of many forms of congenital deafness (compared to inherited irreversible vision loss) counteracts some of this benefit. In studies with mice, this issue can be partially overcome by the fact that the sensory organs of the eye and ear are not fully developed at birth, and thus therapy can be administered very early in the course of the pathology. However, this advantage is lost in the transition to human patients. Individuals with an early developmental disorder would need to have therapy administered while still in the womb, which introduces additional challenges to the already complex nature of cochlear gene delivery. Regardless of the time-of-onset, early detection and intervention in patients is key to effective treatment. Clinical support for this idea comes from early reports from the LCA retinal gene therapy trials. RPE65-associated LCA is an early onset disease (in contrast to many inherited retinal diseases), and in the initial phase 1 clinical trial the greatest improvement was observed in the younger children (Maguire et al., 2008, 2009). The need for intervention early in the disease process also highlights the need for treatments and delivery strategies that do not themselves result in vision or hearing loss. Both subretinal injection and cochlear delivery methods have the potential to impair function (Wang et al., 2013), so optimizing delivery approaches should remain a key research goal.

In addition to delivery at the appropriate time in the course of the disease pathology, an additional concern is the potential need for repeat dosing. The ideal gene therapy would be effective long-term after delivery of a single dose. However, generating and maintaining sufficient levels of gene expression remains a challenge. Improving duration of expression at levels high enough to maintain functional rescue is essential (Adijanto and Naash, 2015; Hardee et al., 2017; Goswami et al., 2019; Patil et al., 2019; Wang et al., 2019).

In the case of the retina, the current measures of therapeutic longevity come from the early LCA trials, where results have been variable. Some groups reported functional visual improvement up to 3 years despite cell degeneration still taking place (Cideciyan et al., 2013; Testa et al., 2013), while other groups reported that the improvement in vision diminished in patients after 3 years (Bainbridge et al., 2015; Jacobson et al., 2015). In other cases, it has been reported that the beneficial effects are more durable, persisting at the 4 year follow-up (Maguire et al., 2019) however, different trials have used different outcome measures to assess improvement. The mechanism underlying limited duration of effects is unclear, however it has been suggested that the virally-expressed RPE65 levels declined over time to below the levels necessary to remain therapeutically relevant (Bainbridge et al., 2015). It is also possible that loss of non-transduced RPE cells led to such severe stress in the retina that the transduced RPE cells were insufficient to prevent the eventual photoreceptor degeneration. Clearly, these findings highlight both the promise and vast room for continued technical and scientific advancement needed for clinical application of retinal and cochlear gene therapy.

## Future Perspectives

Both the eye and cochlea are organs which have favorable anatomy for gene therapy with small sizes that can be easily treated with small doses of therapeutics, a contralateral control and easy non-invasive analytical techniques. They are also sites of many different forms of inherited hearing loss and irreversible vision loss caused by several hundred known genes, and thus they are prime targets for novel therapies. The monogenic nature of most inherited diseases of the eye and ear make gene therapy an attractive method to potentially correct causative genetic defects and cure disease, rather than just treating symptoms. There are various limitations and challenges still to face including (1) the limited number of patients carrying any given mutation, although allele-independent approaches are being developed, (2) limited carrying capacity of common viral-delivery methods, (3) the rapid progression of degeneration in some pathologies, (4) limited distribution and longevity of gene expression after single-dose treatments, (5) difficulty transfecting/transducing post-mitotic cells, and (6) the need for delivery methods that are less invasive. Yet all these areas are being actively studied, and outcomes with genetic therapies are likely to keep improving. Another area that has seen rapid progress in the last decade is genetic diagnosis. The accessibility and accuracy of genetic testing in the past few years has improved drastically (Shearer et al., 2011; Lin et al., 2012; Furutani and Shimamura, 2017; Men et al., 2017; Furutani et al., 2019). As genetic testing and screening improve, the ability to identify causative pathogenic alleles and potential at-risk individuals increases. Earlier screening and diagnosis are essential to earlier intervention.

The commonalities between the eye and the ear, coupled with the numerous syndromic genetic disorders with both an auditory and visual component mean that coordination between the fields is likely to be beneficial. Collaboration and resource sharing between groups studying both the retina and the cochlea may help advance the development of clinically relevant therapies for both vision and hearing loss, and lead to significantly improved quality of life in patients.

## Author Contributions

RC wrote initial draft and created the figures. SMC, MRA, and MIN were editors of final draft. All authors contributed to the article and approved the submitted version.

## Conflict of Interest

The authors declare that the research was conducted in the absence of any commercial or financial relationships that could be construed as a potential conflict of interest.

## Footnotes

1 https://www.who.int/en/news-room/fact-sheets/detail/blindness-and-visual-impairment

2 https://www.who.int/en/news-room/fact-sheets/detail/deafness-and-hearing-loss

3 https://www.cdc.gov/visionhealth/basics/ced/index.html

4 https://hereditaryhearingloss.org/

5 https://clinicaltrials.gov/
